# *In vitro* characterization of antimicrobial efficacy and cytotoxicity of polyvinylpyrrolidone-iodine, N-acetylcysteine, methylglyoxal, and N-chlorotaurine as alternative antimicrobials in treating bovine clinical endometritis

**DOI:** 10.3389/fvets.2025.1699857

**Published:** 2026-01-16

**Authors:** Dijana Pas, Hilke Oltmanns, Jessica Meißner

**Affiliations:** Department of Pharmacology, Toxicology and Pharmacy, University of Veterinary Medicine Hannover, Foundation, Hannover, Germany

**Keywords:** bovine endometritis, alternative antibacterials, cytotoxicity, *Trueperella pyogenes*, primary cell culture

## Abstract

Bovine clinical endometritis (CE) is a common indication for antibiotic use in dairy cows. The increase in bacterial resistance and the aspired decrease in antibiotic use under the One Health concept call for alternatives in treatment. Polyvinylpyrrolidone-iodine (PVP), N-acetylcysteine (NAC), methylglyoxal (MGO), and N-chlorotaurine (NCT) are known substances with antibacterial properties that could potentially serve as those alternatives. In a broth microdilution assay, their efficacy against the common cause of endometritis, *Trueperella pyogenes*, was investigated. By cytotoxicity testing on a primary bovine endometrial epithelial cell culture, potential adverse effects on cell proliferation, viability, and immune response (IL-6) were examined. While all four substances had an antibacterial effect on *T. pyogenes*, PVP, MGO, and NCT also showed cytotoxic effects. In contrast, NAC was tolerated well by the cells. In sum, the four tested substances can be considered potential alternatives to antibiotic treatments. Further research is, however, necessary to investigate their toxic effects in *ex vivo* or *in vivo* models and to identify effective dosages in animals.

## 1 Introduction

Bovine clinical endometritis (CE) is defined by Sheldon et al. (1) as an illness showcasing mucopurulent or purulent uterine discharge 21 days postpartum or later. It is not accompanied by any systemic clinical symptoms. In addition to uterine discharge, endometrial inflammation is a key component of CE (2). Cows with CE suffer not only from the illness itself, which lowers animal welfare, but also from higher rates of infertility or subfertility, which lead to economic losses for the farmer and increased culling of affected animals (3). The pathogenesis of CE involves three major pathogenic pathways: alterations in immune function (4, 5), metabolic stress (6, 7), and shifts in the uterine microbiome (8, 9). For the latter, various pathogens have been identified as playing a role in CE. One of the most common bacteria is *Trueperella pyogenes*, which has been directly linked to a higher risk of CE 21 days postpartum (10). *T. pyogenes* is a Gram-positive, opportunistic rod that causes purulent and necrotic lesions (11). Its pathogenicity in the endometrium stems from its pathogenic factor, pyolysin, which disrupts the endometrial barrier by its cytolytic activity and activates an inflammatory process in the endometrium (12). Routine treatment of CE includes parenteral application of prostaglandins or intrauterine application of antibiotics, for example, cephapirin (13). However, due to emerging antimicrobial resistance, there is a need for alternative treatment options or for reducing antibiotic use (14).

One already applied alternative is intrauterine infusion with diluted polyvinylpyrrolidone-iodine (PVP) (15). However, it is not entirely clear whether this treatment is fully beneficial. While *in vitro* studies show significant cytotoxic effects (16), *in vivo* testing on successful insemination rates suggests only transient inflammation of the uterus with subsequent benefits to the endometrium (17, 18). It shows antimicrobial properties through the biochemically active, free I_2_, which maintains an equilibrium with the I_2_ bound to polyvinylpyrrolidone (19). I_2_ acts oxidatively at different sites in the bacterium, which denatures and deactivates affected biomolecules and makes it hard for resistance to develop due to the broad possibility of points to attack (20, 21).

In equine medicine, some studies have examined the efficacy of N-acetylcysteine (NAC) in mares with endometritis. While endometrial integrity was preserved, there were no significant effects of intrauterine NAC-infusion on fertility (22). NAC originates from the acetylation of the amino acid L-cysteine and has many mechanisms of action, such as scavenging oxidants, replenishing glutathione, reducing disulfides, and, more recently, sulfane–sulfur effects (23). Various studies have shown its antibacterial and antibiofilm properties in ophthalmology, odontology, and against general pathogens on the skin (24–27).

Methylglyoxal (MGO) is an active component of manuka honey and its leading antibacterial agent (28). Manuka honey is exclusively produced by the nectar of *Leptospermum scoparium*, a plant that is native to New Zealand and Southeast Australia, and shows extraordinarily high contents of MGO (29). Many studies show MGO’s ability to inhibit the growth of different bacteria, including multidrug-resistant bacteria (30–32). It unfolds its antibacterial properties by disrupting the cell membrane of both Gram-positive and Gram-negative bacteria and inhibiting the formation of fimbriae and flagella (33). Manuka honey finds its use in veterinary medicine as an additive to wound dressings to promote wound healing and avoid infection (34).

N-chlorotaurine (NCT) is a derivative of the amino acid taurine, which is produced in the body by granulocytes and monocytes during the respiratory burst (35). It has also been synthesized as a sodium salt, which shows benefits in handling (35). Anich et al. showed NCT’s ability to inhibit the growth of multidrug-resistant bacteria and proved its efficacy was as high as against non-resistant bacteria (36). The evasion of resistance is due to the oxidizing and chlorinating effects that NCT has on multiple bacterial targets (37).

In this study, the antibacterial efficacy and cytotoxic effects of PVP, NAC, MGO, and NCT were evaluated against *T. pyogenes* and primary bovine endometrial epithelial cells to screen these substances for concentrations that are both effective and tolerable, since they have previously shown antibacterial properties. Potential concentrations serve as the basis for further *ex vivo* and/or *in vivo* experiments to identify applicable alternative treatments for bovine clinical endometritis, thereby minimizing or even excluding antibiotic use.

## 2 Materials and methods

### 2.1 Ethics statement

In Germany, this project does not require ethical clearance because the organs used were obtained from an abattoir. No animals were used or killed for the purpose of obtaining organs or bacteria. They were slaughtered for consumption, and the uteri made available were a byproduct of this process.

### 2.2 Bacterial isolation

Next to the reference bacterium *T. pyogenes* DSM 20594 from the Leibniz Institute DSMZ-German Collection of Microorganisms and Cell Cultures GmbH, *T. pyogenes* isolates were obtained by sampling uteri from a local abattoir. When a uterus showed signs of inflammation and mucopurulent or purulent discharge upon opening its horns with sterile scissors, a cotton swab was taken out of each horn individually and used for a fractioned smear on Columbia sheep blood agar (Oxoid, Wesel, Germany). After 24 h of incubation at 37 °C and 5% CO_2_, colonies morphologically similar to *T. pyogenes* were subcultured on Columbia sheep blood agar for an additional 24 h. These subcultures were identified using matrix-assisted laser desorption/ionization–time of flight (MALDI–TOF). When *T. pyogenes* was identified, it was frozen and stored at −70 °C. Eight isolates were gained using this method.

### 2.3 Microdilution assay

For the microdilution assay, *T. pyogenes* was grown on Columbia sheep blood agar for 48 to 72 h and suspended in 0.9% saline solution at an optical density of 1.0 McFarland units. The test substances were dissolved and diluted in Micronaut-H-medium (Bruker, Bremen, Germany) at concentrations of 5.0–6.75 mg/mL for PVP (Sigma–Aldrich, Steinheim, Germany; 0.25 mg/mL per dilution step), 2.0–2.7 mg/mL for NAC (Sigma–Aldrich, Steinheim, Germany; 0.1 mg/mL per dilution step), 0.1–0.8 mg/mL for MGO (Sigma–Aldrich, Steinheim, Germany; 0.1 mg/mL per dilution step), and 1.0–3.5 mg/mL for NCT (Alchemist, Stuttgart, Germany; 0.25 mg/mL per dilution step). On a U-bottom 96-well plate (Greiner, Frickenhausen, Germany), 90 μL of medium with test substance or pure medium were mixed with either 10 μL of bacterial suspension (for positive control in medium and for the testing in medium with diluted test substance) or 10 μL of saline solution (negative control for medium and medium with test substance). After an incubation time of 48 h, the first concentration of test substance that showed no bacterial growth by visual inspection was considered the minimal inhibitory concentration (MIC) as defined by the Clinical and Laboratory Standards Institute (CLSI). For each of the nine isolates, the microdilution assay was repeated 6 times in biological replicates.

### 2.4 Bovine endometrial epithelial cell isolation

Bovine endometrial epithelial cells (BEEC) were isolated using a modified protocol from Nongbua et al. (38). Uteri from at least 24-month-old Holstein Friesian cows were obtained from a local abattoir and transported for approximately 2 h on ice. In the laboratory, the uteri were examined to rule out macroscopic infection or inflammation, and the cycle stages were determined by reviewing the ovaries as previously described (39). Only healthy uteri in the cycle stage I were used for further preparations. First, both uterine horns were opened with sterile scissors, and the endometrium was cut out into small pieces (approximately 3 mm) utilizing sterile small forceps and scissors. The endometrial pieces were transferred into digestion medium containing 250 U/mL collagenase IV (Sigma–Aldrich, Steinheim, Germany), 1,000 U/mL hyaluronidase (Roth, Karlsruhe, Germany) and 2% bovine serum albumin (BSA, Sigma–Aldrich, Steinheim, Germany) in Dulbecco’s phosphate buffered solution (DPBS, Roth, Karlsruhe, Germany) and digested for 2.5 h in a water bath at 39 °C. Every 30 min, the suspension was vortexed for 1–2 s. After incubation, the suspension was filtered using, first, gaze to remove larger tissue chunks, and then a 100μm filter (Sarstedt, Nümbrecht, Germany) for filtering the cell suspension. The suspension was then centrifuged for 5 min at 150 *g* and resuspended in culture medium containing 10% fetal calf serum (Bio&SELL, Feucht, Germany), 5ml insulin–transferrin–selenium (ITS, Gibco, Carlsbad, CA, USA), 50 U/mL penicillin (Gibco, Carlsbad, CA, USA), 50 μg/mL streptomycin (Gibco, Carlsbad, California, USA), 10 μg/mL gentamicin (Roth, Karlsruhe, Germany), and 2,5 μg/mL amphotericin B (Bio&SELL, Feucht, Germany) in Dulbecco’s modified eagle medium + Ham’s 12 medium (DMEM F12) supplemented with 2mM glutamine (Roth, Karlsruhe, Germany). The cell suspension was transferred into a 75 cm^2^ cell culture flask (Greiner, Frickenhausen, Germany) and incubated at 37 °C and 5% CO_2_.

To ensure pure epithelial cell cultures, the method of selective passaging was adapted from Kelly et al. (40). BEEC were passaged at about 90% confluency by first detaching stromal cells with accutase (Bio&SELL, Feucht, Germany) for 5 min, washing them out with DPBS and discarding them, and then detaching the epithelial cells with 0.25%/0.02% trypsin/ethylenediaminetetraacetate (EDTA, Biochrom, Berlin, Germany) for about 10 min and seeding them at 100,000–500,000 cells per flask. At passage 3, the culture was almost free of stromal cells as proven by fluorescent immunocytochemistry.

### 2.5 Fluorescent immunocytochemistry

To identify the cells as either epithelial or stromal, fluorescent immunocytochemistry was performed on each isolation at passage 3 or higher. In four wells of a 24-well plate (Greiner, Frickenhausen, Germany), sterile glass coverslips (Roth, Karlsruhe, Germany) were placed, and 10,000 cells/well were seeded onto them. Each well will have one of the following staining: (1) cytokeratin, (2) vimentin, (3) a combination of cytokeratin and vimentin, and (4) negative control. The following day, the cell culture medium was removed, and the wells were washed twice with 300 μL/well phosphate-buffered solution (PBS). Then, cells were fixated with 300 μL/well of an ice-cold 1:1 mixture of acetone (Sigma–Aldrich, Steinheim, Germany) and methanol (Avantor Performance Materials Poland, Gliwice, Poland) for 5 min. As a next step, cells were blocked at room temperature with 300 μL/well of a blocking solution consisting of 0.25% Triton X-100 (Sigma–Aldrich, Steinheim, Germany) and 1% BSA in PBS. After removing the blocking solution, the first antibody, a primary mouse pan-anticytokeratin (C11; Novus Biologicals, Wiesbaden, Germany), was diluted 1:100 in the blocking solution and added to wells 1 and 3 at 150 μL/well. Wells 2 and 4 were blocked by 150 μL/well blocking solution. Following a 1h incubation at 37 °C, all wells were washed twice with 300 μL/well PBS for 5 min. The second antibody, a fluorescein-marked secondary goat F(ab)2-anti-mouse-antibody (AbD serotec, Puchheim, Germany), was diluted 1:200 in blocking solution and added to wells 1, 3, and 4 at 150 μL/well. To well 2, a 150-μl blocking solution was added and the plate incubated at 37 °C for 30 min. All wells were washed twice after the first antibody. After the third antibody, a cyanin3-marked monoclonal mouse anti-vimentin antibody (Sigma, Steinheim, Germany), was diluted to 1:200 in blocking solution and added to wells 2 and 3 at 150 μL/well. Well 1 and 4 were blocked by 150 μL/well blocking solution, the plate was incubated at 37 °C for another 30 min, and all wells were washed twice as in previous steps. At last, the coverslips were attached to microscope slides and cell nuclei were stained using Fluoroshield™ DAPI (Sigma, Steinheim, Germany). The staining was evaluated by fluorescent microscopy with the Zeiss ObserverZ1/1 microscope and Zeiss Zen 3.8 software (Carl Zeiss Microscopy, Oberkochen, Germany).

### 2.6 Cytotoxicity assays

For cytotoxicity testing, BEEC were seeded onto F-bottom 96-well-plates (Greiner, Frickenhausen, Germany) at a density of 10,000 cells/well in culture medium. Treatments included PVP (5.75 and 6 mg/mL), NAC (2.4 and 2.5 mg/mL), MGO (0.4 and 0.5 mg/mL), and NCT (2.25 and 2.5 mg/mL), which were dissolved and diluted in culture medium, and pure culture medium as a control. These concentrations were chosen after an initial screening of MIC of the reference bacterium and represent the median MIC itself as well as one concentration just above the MIC. For the neutral red assay and bovine interleukin 6 (IL-6) enzyme-linked immunosorbent assay (ELISA) cells were also treated with 1 μg/mL lipopolysaccharide O111: B4 (LPS, from *Escherichia coli*, Sigma–Aldrich, Steinheim, Germany) as well as 1 and 10 μg/mL peptidoglycan (PGN, from *Staphylococcus aureus*, Sigma–Aldrich¸ Steinheim, Germany). Both the crystal violet staining and the neutral red uptake assay were undertaken in six biological replicates, each representing an independent cell isolation from different animals, and eight technical replicates. Relative proliferation and viability values were calculated by the following equation (Equation 1):


$$\begin{array}{l} \text{Percentage}\;(\%)\;\text{of control}=\\ \;\;\;\;\;\;\;\;\;\;\frac{{\mathrm{OD}}_{570\;\mathrm{nm}}\;\text{of well}\times 100}{\text{mean}\;{\mathrm{OD}}_{570\;\mathrm{nm}}\;\text{of control wells}}\; \end{array}$$ (1)

#### 2.6.1 Crystal violet staining assay

To determine cell proliferation, a modified crystal violet staining assay (41, 42) was performed. Notably, 24 h after seeding, cells were treated as described above with 100 μL of each solution per well and incubated for an additional 24 h. After removal of the treatments, cells were fixated for 20 min using 100 μL/well 2% glutaraldehyde (Roth, Karlsruhe, Germany) in PBS and stained with 100 μL/well 0.1% crystal violet dye (Roth, Karlsruhe, Germany) in distilled water for 30 min. After washing the wells with 300 μL/well distilled water and air-drying for about 2 h, crystal violet was extracted for 1 h on an orbital shaker (SO3, Stuart Scientific, Stone, Great Britain) at 250 rpm using 50 μL/well 2% tergitol in distilled water (Sigma–Aldrich, Steinheim, Germany) and the optical density was measured in an MRX-reader (Microplate-reader Multiscan FC, Thermo Scientific, Waltham, MA, United States) at 570 nm. Each step after incubation was performed at room temperature.

#### 2.6.2 Neutral red uptake assay

To determine cell viability, a neutral red uptake assay was performed. Notably, 72 h after seeding, cells reached confluency and were treated as mentioned above for 24 h. Supernatants were collected and stored at −20 °C for later usage in a bovine IL-6 ELISA. Neutral red stock solution (1 mg/mL in distilled water, AppliChem, Darmstadt, Germany) was diluted 1:100 in cell culture medium and incubated at 37 °C for 30 min. After centrifuging for 10 min at 1,500 *g*, 200μl neutral red solution was added to each well, and the plate was incubated for 3 h in the incubator. After removal of the neutral red solution, the plate was laid upside down for approximately 2 min to dry. Then, 100 μL of a solution containing 1% calcium chloride (Merck, Darmstadt, Germany) and 0.5% formaldehyde (Merck, Darmstadt, Germany) in distilled water was added to each well and removed. At last, 100 μL of a solution consisting of 1% acetic acid (AppliChem, Darmstadt, Germany) and 50% ethanol (CG-Chemikaliengesellschaft, Laatzen, Germany) in distilled water was added to each well and shaken on an orbital shaker for 30 s. After an incubation in the fridge for 10 min, the optical densities were measured using an MRX-reader at 570 nm. The assay was executed according to the International Organization for Standardization (ISO) DIN Norm 10,993–5-2009(E).

#### 2.6.3 Bovine IL-6 enzyme-linked immunosorbent assay

For the bovine IL-6 ELISA, the DuoSet™ ELISA Development System with additional DuoSet™ Ancillary Kit 3 (R&D Systems, Minneapolis, Minnesota, United States) was used according to the manufacturer’s protocols. Supernatants were collected from confluent cells after 24 h of treatment and frozen at −20 °C. The supernatants from the LPS treatment were diluted to 1:10.

### 2.7 Statistical analysis

All statistical analyses were performed using the GraphPad Prism Software (version 10.4.1 (627), GraphPad Software, Inc., Boston, MA, United States). Descriptive statistics were applied to the MIC values of all *T. pyogenes* isolates. Cytotoxicity data were analyzed using a Friedman test followed by Dunn’s multiple comparisons test comparing each treatment with the culture medium control. Differences were considered statistically significant at *p* ≤ 0.05.

## 3 Results

### 3.1 Minimal inhibitory concentration

For all four substances (PVP, NAC, MGO, and NCT), the MIC values of the reference bacterium and eight *T. pyogenes* isolates were determined. Altogether, the median MIC value for PVP was at 5.75 mg/mL, with a range of 1.5 mg/mL and an interquartile range (IQR) from 5.5 to 5.75 mg/mL. For NAC, the median was at 2.5 mg/mL with a range of 0.7 mg/mL and an IQR from 2.4 to 2.6 mg/mL. MGO showed a median MIC of 0.2 mg/mL with a range of 0.3 mg/mL and an IQR from 0.2 to 0.3 mg/mL. Finally, NCT had a median MIC of 2 mg/mL with a range of 1.5 mg/mL and an IQR from 1.75 to 2.25 mg/mL.

### 3.2 Fluorescent immunocytochemistry

**Figure 1:**
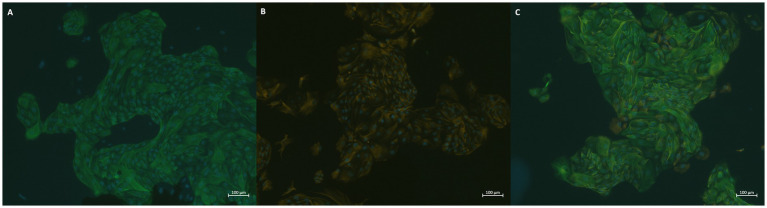
BEECs were stained with **(A)** FITC (green) for epithelial cells, **(B)** Cy3 (orange) for stromal cells individually, as well as **(C)** on the same slide, and DAPI (blue) for cell nuclei on all slides.

In addition to morphological control in light microscopy, all cell isolations used for cytotoxicity testing were stained by fluorescent immunocytochemistry to ensure that only epithelial cells were included in testing. Staining was performed at passage 3 or 4 and resulted in the identification of pure epithelial endometrial cells, as exemplified by Figure 1.

### 3.3 Cytotoxicity assessment

**Figure 2:**
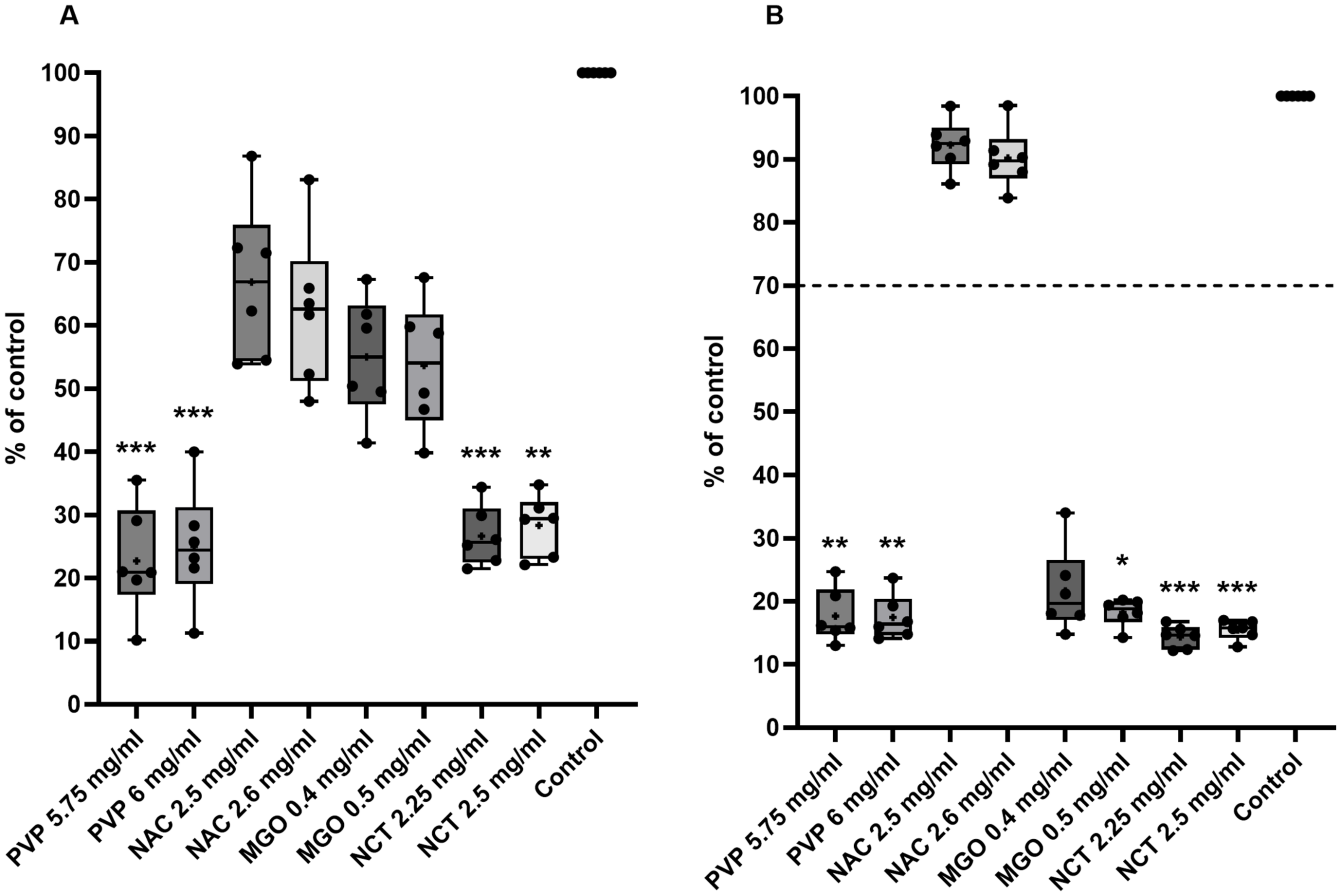
Influence of two concentrations of each tested substance on **(A)** proliferation and **(B)** viability of BEEC. The *x*-axis shows antibacterial substances in their tested concentrations; the *y*-axis shows cell proliferation/viability as the percentage of control cells. The data is presented as boxplots with min/max-whiskers, dots stand for the mean of each biological replicate (tested in an 8-fold technical replicate), and the overall mean is indicated by a +. The dotted line represents the 70% limit for cytotoxicity testing according to ISO 10993-5:2009. Data were analyzed by the Friedmann test following Dunn’s multiple comparisons test of each treatment with the culture medium control. Significant differences between treated and control cells are depicted by asterisks: *p ≤ 0.05; ** ≤ 0.01; *** ≤ 0.001. *n* = 6.

In proliferation and viability testing, PVP, NAC, MGO, and NCT generated the following effects on BEEC compared with cells treated with culture medium as the control (Figure 2). Only treatment with PVP and NCT at their respective concentrations resulted in significantly lower proliferation and viability compared to control cells. Additionally, MGO significantly reduced cell viability at a concentration of 0.5 mg/mL.

The cells treated with PVP showed a proliferation of 22.7 ± 8.7% at a concentration of 5.75 mg/mL and 25.0 ± 9.4% at a concentration of 6.0 mg/mL. The viability values were 17.7 ± 4.3% and 17.5 ± 3.6%, respectively. When cells were treated with 2.5 and 2.6 mg/mL NAC, proliferation compared to control was 66.9 ± 12.6% and 62.4 ± 12.2% and viability was 92.3 ± 4.1% and 90.2 ± 4.8%. The cells treated with 0.4 and 0.5 mg/mL MGO showed proliferation values of 55.0 ± 9.5% and 53.7 ± 10.2%, respectively, whereas viability decreased to 21.7 ± 6.8% and 18.3 ± 2.2%. NCT at the concentrations of 2.25 and 2.5 mg/mL reduced proliferation to 26.7 ± 4.8% and 28.4 ± 4.8% and viability to 14.4 ± 1.8% and 15.5 ± 1.5%.

### 3.4 IL-6 release

**Figure 3:**
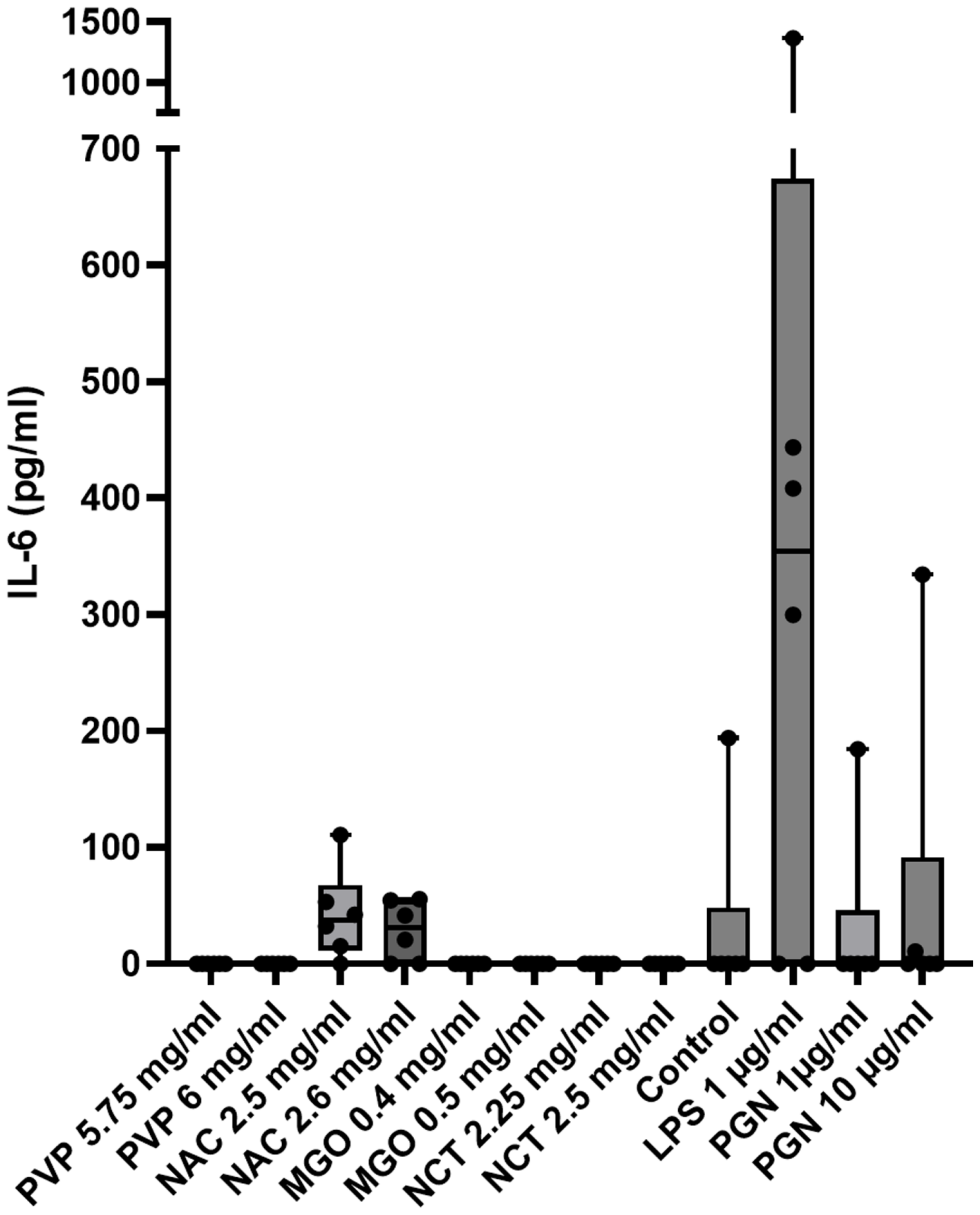
IL-6 release of two concentrations of each tested substance, as well as LPS at 1 μg/mL, PGN at 1 μg/mL and 10 μg/mL, and control (medium). The *x*-axis shows all treatments in their tested concentrations; the *y*-axis shows the release of IL-6 in pg/ml. The data are presented as boxplots with minimum/maximum whiskers, and dots represent each measured value in the bovine IL-6 ELISA assay (six biological replicates). Data were analyzed by the Friedman test following Dunn’s multiple comparisons test for each treatment with the culture medium control and LPS 1 μg/mL. No significant differences could be detected. *n* = 6.

Results of the bovine IL-6 ELISA show that, in contrast to NAC (50.9 ± 36.3 for 2.5 mg/mL; 43.1 ± 16.2 pg./mL for 2.6 mg/mL) treatment, with PVP, MGO, and NCT led to no detectable release of IL-6 (Figure 3). Treatment with LPS resulted in IL-6 release up to 1,366 pg/mL (629 ± 495 pg./mL). Cells treated with cell culture medium as a control released no detectable IL-6 except for one biological replicate, which released 193.9 pg./mL. There were no significant differences between the control and the treatments, or between 1 μg/mL LPS and the treatments.

## 4 Discussion

In this study, *T. pyogenes* was isolated from organs of slaughtered cows. While organs were selected based on apparent inflammation and discharge, it is hard to determine whether these findings were clinically relevant to the affected cow. However, *T. pyogenes* was found mostly in cows with CE in comparison to healthy cows or cows with subclinical endometritis (43). Considering this, any involvement of *T. pyogenes* would be significant for treating a possible inflammation, and any isolated *T. pyogenes* would yield results applicable to studying MICs.

The only substance that has been tested with *T. pyogenes* for its MIC before is PVP (16). The MIC value was 1.25% in a broth microdilution with tryptone soy broth, which is more than twice the value reported in this study. This could be due to differences in setups used across the tested concentrations. While Thongrueang et al. used 10-fold serial dilutions, this setup included 0.25 mg/mL steps, screening for a more precise MIC rather than a general direction, which, in this case, would support the conclusion that the findings do not refute one another. In contrast, Mido et al. (15) found concentrations of 0.5 and 2.0% PVP to be effective against bacteria from the uterus *in vitro* as well as *in vivo*.

While there was no published research found on the effect of NAC against *T. pyogenes*, there are studies on its efficacy against other pathogens, such as different *Staphylococcus* and *Streptococcus* spp., *E. coli*, *Pseudomonas aeruginosa*, and *Klebsiella pneumoniae* (24, 44–46). Overall, MIC ranged from 1.56 to 10 mg/mL, indicating that NAC has sufficient antibacterial activity at those concentrations. Our findings fall within the range of previous studies at 2.5 mg/mL for *T. pyogenes*, and therefore can be considered to be realistic results. In addition, Bohn et al. even found the same MIC at 2.5 mg/mL for two multiresistant and one sensitive isolate of *Staphylococcus pseudintermedius* (47).

MIC determination for MGO was also not previously performed for *T. pyogenes*. A study conducted on multidrug-resistant *P. aeruginosa* in agar dilution testing observed MIC values for methylglyoxal of 128–512 μg/mL (30). Green et al. detected similar concentrations in a broth microdilution assay with *S. aureus*, *Enterococcus faecalis*, *E. coli*, and *P. aeruginosa* (28) and showed that honey’s antibacterial properties correlate with its MGO content. In this study, MGO concentrations of 0.2 mg/mL were found to inhibit bacterial growth, making them comparable to the aforementioned studies.

There is currently no data available on any MIC for NCT. However, in biofilm reduction studies, 1% NCT showed a significant reduction of biofilms (48, 49). In this study, NCT was effective against *T. pyogenes* at lower concentrations (2 mg/mL), likely because the antibacterial substances function more effectively when they do not have to breach a biofilm first.

Overall, the concentrations were determined by broth microdilution in a medium specifically adapted for *T. pyogenes*. It is to be expected that under these *in vitro* conditions, different results are obtained than in *in vivo* testing, which is why concentration adaptation needs to happen in animal experiments. *In vitro* testing, however, shows a general direction for the applicability of the tested substances, reducing animal testing to only the most promising substances and concentrations.

Concentrations for cytotoxicity testing were chosen based on preliminary MIC results to show that effective antibacterial concentrations could be tolerated by the endometrial epithelium. PVP has already been shown to be cytotoxic at low concentrations (50). For bovine endometrial epithelial cells, only 1 h of exposure to 0.025% PVP led to a significant loss of viability (16). In this study, PVP was again tested to undermine the cytotoxic effect of PVP, since in practice it is still commonly used as a diluted irrigation to flush the uteri of cows with CE (15). In a study on corneal toxicity, Chen et al. found one mechanism of cytotoxicity of PVP to be an increase in oxidative stress (51). Also studying corneal toxicity, Chou et al. saw cytotoxicity mainly due to the halt of intracellular esterase activities and lowered cell membrane integrity (52).

In mares, intrauterine administration of NAC (5 and 3.3%) did not result in any adverse effects on the endometrium (22, 53). In this study, concentrations of 2.5 and 2.6 mg/mL were much lower than those tested *in vivo* and, as expected, were tolerated well by the BEEC. Especially for NAC, further research on *ex vivo* and *in vivo* models seems therefore promising. Another aspect that could be studied is the possible mucolytic effects on uterine discharge.

The effects of MGO on BEEC have not been studied. However, it showed cytotoxic effects against tumor cells due to oxidative stress, resulting in apoptosis, which made it an interesting topic in anti-tumor therapy (54, 55). But there are many more pathways of cytotoxicity to be found in the literature, for example, dysfunction of SERCA pumps, mitochondria-dependent apoptosis, and endoplasmic reticulum stress-associated production of reactive oxygen species (56–60). The tested concentrations in this study confirm cytotoxicity in BEEC as well. Since Manuka Honey itself does not show such drastic cytotoxic effects, with methylglyoxal only being one of its many biologically active components, further research into Manuka Honey would be interesting, even with the challenges of standardization in this natural product (29).

In this study, NCT was not well tolerated. This stands in contrast to previous studies conducted on different cell lines, which reported good tolerability of NCT at concentrations of 0.01–1% (61, 62). While the *in vitro* cytotoxicity results in this study indicate that NCT might not be a well-suited antibacterial alternative, other research shows that NCT was often tolerated better *in vivo* than it was *in vitro* (35, 61, 63). This could have different reasons, such as a more robust barrier of cells built *in vivo*, protection by bodily fluids or mucus, or shorter exposure times. Considering this, NCT seems a noteworthy candidate for further testing in its antibacterial concentrations.

IL-6 is a pro-inflammatory cytokine in the context of CE (4). Therefore, the effects of PVP, NAC, MGO, and NCT on their release were investigated to draw conclusions on whether these substances could modulate the immune response in the endometrium. Treatment with PVP, MGO, and NCT did not result in detectable IL-6 in the collected supernatants. However, it must be noted that the concentrations used were already cytotoxic and therefore the release of cytokines could not be expected. When treated with NAC, cells had a higher survival rate, which explains the higher IL-6 concentration in those supernatants compared to the other treatments. Due to the high variability in IL-6 release in the positive control with LPS, it is hard to contextualize this IL-6 concentration after treatment with NAC. However, it also did not induce a significantly higher release of IL-6 than the control, which suggests no significant proinflammatory response was mounted after NAC treatment. Previously, NAC has shown anti-inflammatory effects (23, 64), so the IL-6 release could be non-significant compared to properly mounted reactions to a positive control. This limitation, arising from two biological replicates, showing no detectable IL-6, could have several explanations. While IL-6 is a relatively stable analyte, there are conflicting study results on optimal storage temperature and the number of possible freeze/thaw cycles (65). A problem with its stability in these particular replicates could therefore be one explanation. Another explanation could be the lack of predictability of the primary cell culture in this *in vitro* model. Even though this was not a problem when investigating proliferation and viability, intracellular pathways might be inadequate to mount a reliable immune response. Cytotoxicity, as an explanation for the absence of detectable IL-6, can be ruled out, as the concentrations of LPS and PGN used were tested for tolerability by the cells before conducting the ELISA.

One challenge in this study was the very low success rate in endometrial epithelial cell isolations. Only 25% of all isolations provided pure and viable epithelial cell culture that could be passaged up to 6 times or more, and were taken into consideration for cytotoxicity testing. Failed isolations were mostly due to stromal cells outgrowing the epithelial cells irretrievably or epithelial cell death before reaching passage 3. Only successful cell isolations could be used for the cytotoxicity assays, thus providing reliable data. For future experiments, a refined isolation method would be essential if a high screening rate is required.

In summary, the results of this research provide greater insight into four known antibacterial substances for the treatment of bovine clinical endometritis. All of them performed well in broth microdilution assays against *T. pyogenes*. However, more testing by, for example, time-killing assays or determination of minimal bactericidal concentrations would improve possible dosages that could be screened in animal experiments. Another interesting scope would be the effect of these substances on the endometrial immune response and on the activation of intracellular cascades, as has been reported for other cells (66). In addition, infection assays with an *ex vivo* endometrial model would provide a better understanding of possible protection of tissue against bacterial infection, possibly outweighing the cytotoxic effects shown for PVP, MGO, and NCT when tissue is left in a unit, as would be the case for endometrial explants (67). For future practical relevance, all substances could be used as an easily applicable solution for intrauterine infusion and flushing, as is already the case for PVP (15). For those, stability testing would be necessary before commercial availability. Another approach could be the combination with classic antibiotics to identify possible synergistic and/or antagonistic effects (68–71).

## 5 Conclusion

All tested alternatives to antibiotics appear applicable based on these *in vitro* results, given their antibacterial efficacy against *T. pyogenes*. However, the cytotoxic effects of PVP, MGO, and NCT on BEEC show that more research is needed to determine whether those effects translate into an *ex vivo*/*in vivo* setting and would therefore eliminate those substances as viable intrauterine treatment options.

## Data Availability

The raw data supporting the conclusions of this article will be made available by the authors, without undue reservation.
